# Self-perceptions of aging, race-based discrimination, and total cognition among diverse older adults

**DOI:** 10.1093/geroni/igaf122.3651

**Published:** 2025-12-31

**Authors:** Andrew Steward, Cliff Whetung, Yura Lee, Tyrone Hamler

**Affiliations:** University of Wisconsin-Milwaukee, Milwaukee, Wisconsin, United States; University of Minnesota Duluth, Duluth, Minnesota, United States; University of Wisconsin-Milwaukee, Milwaukee, Wisconsin, United States; University of Denver, Denver, Colorado, United States

## Abstract

Research drawing from stereotype embodiment theory suggests that positive self-perceptions of aging (SPA) are associated with enhanced cognitive function among older adults. While everyday discrimination is associated with negative SPA and reduced cognitive function, the relationship between race-based discrimination, SPA, and cognitive function remains unknown. Therefore, we drew from the Health and Retirement Study to examine associations between SPA, race-based discrimination, and cognition among a racially diverse sample of adults 50+ years of age (N = 5,949) between 2014 and 2020. We computed weighted descriptive statistics and estimated mixed-effect regression models, adjusting for theoretically informed covariates. Among respondents who primarily attributed everyday discrimination to race (N = 894), the self-reported racial identity profile was 46.17% Black/African American, 40.73% White, 9% Asian, and 4.53% Indigenous, with 18.19% reporting Hispanic ethnicity. Those who primarily attributed discrimination to race reported the highest standardized everyday discrimination scores (0.72) and lowest standardized SPA scores (-0.19) on average. Overall, more positive SPA was associated with higher cognition (b = 0.49). In mixed-effect models adjusted for upstream predictors of cognition (age, time on study, sex, race, ethnicity, education, income), more frequent experiences of everyday discrimination were associated with lower cognition scores (b=-0.12), but race-based attributions were not associated with differences in cognition. In models stratified by discrimination attributions (no attribution, any race-based attribution, other attributions), SPA was consistently associated with higher cognition scores. These findings may suggest the importance of upstream interventions to promote positive SPA as part of efforts to mitigate the effects of racial discrimination on cognitive health in later life.

